# Supplementary material for: Structural basis of early translocation events on the ribosome
Source: Nature. 2021 Jul 7;595(7869):741–5. doi: 10.1038/s41586-021-03713-x (PMC8318882; doi:10.1038/s41586-021-03713-x)
Supplement: Supplementary file 1 — This file contains Supplementary Tables 1-4 and Supplementary Figure 1. [file 41586_2021_3713_MOESM1_ESM.pdf]

nature portfolio

https://doi.org/10.1038/s41586-021-03713-x

Supplementary information

Structural basis of early translocation events on the ribosome

In the format provided by the authors and unedited

Supplementary Tables

Supplementary Table 1 | Grid preparation, data collection, refinement parameters and model validation.

	**PRE-C** (PDB 7N1P) (EMD 24120)	**PRE-H2*** (PDB 7N30) (EMD 24135)	**PRE-H1** (PDB 7N2U) (EMD 24133)	**POST** (PDB 7N31) (EMD 24136)	**INT1** (PDB 7N2V) (EMD 24134)	**INT2** (PDB 7N2C) (EMD 24132)
Grid manufacturer	Quantifoil	Quantifoil	Quantifoil	Quantifoil	Quantifoil	Quantifoil
Grid type	Holey Carbon	Holey Carbon	Holey Carbon	Holey Carbon	Holey Carbon	Holey Carbon
Grid hole size/spacing	R 1.2/1.3	R 1.2/1.3	R 1.2/1.3	R 1.2/1.3	R 1.2/1.3	R 1.2/1.3
Grid Mesh	300 (Au)	300 (Au)	300 (Au)	300 (Au)	300 (Cu)	300 (Cu)
Sample volume (μl)	3	3	3	3	3	3
Sample concentration (μM)	2	2	2	2	2	2
Blot time (s)	6	6	6	6	6	6
Blot force	-5	-5	-5	-5	-5	-5
Temperature (ºC)	10	10	10	10	10	10
Humidity (%)	95	95	95	95	95	95
Microscope	FEI Titan Krios G3i	FEI Titan Krios G3i	FEI Titan Krios G3i	FEI Titan Krios G3i	FEI Titan Krios G3i	FEI Titan Krios G3i
Voltage (keV)	300	300	300	300	300	300
Camera	K3	K3	K3	K3	K3	K3
Magnification (kx)	105	105	105	105	105	81
Pixel size at detector (Å/pixel)	0.826 (0.413)	0.826 (0.413)	0.826 (0.413)	0.826 (0.413)	0.826 (0.413)	1.06 (0.53)
Total electron exposure (e^{-}/frame*$Å^2$)	87	87	87	87	85	69.8
Exposure rate (e^{-}/pixel/sec)	24.896	24.896	24.896	24.896	21.746	26.144
No. of frames collected during exposure	60	60	60	60	70	60
Defocus range (μm)	-0.4 to -1.5	-0.4 to -1.5	-0.4 to -1.5	-0.4 to -1.5	-0.4 to -1.5	-0.4 to -1.5
Automation software	SerialEM	SerialEM	SerialEM	SerialEM	SerialEM	SerialEM
Energy filter slit width (eV)	20	20	20	20	20	20
Micrographs collected (no.)	7434	7434	7434	3213	12547	7371
Micrographs used (no.)	7183	7183	7183	2834	11916	6651
Total extracted particles (no.)	659,777	659,777	659,777	439,422	1,001,439	125,9307
Refined particles (no.)	534,348	534,348	534,348	159,661	652,128	639,984
Final particles (no.)	109,769	33,330	51,685	113,540	33,688	34,170
Resolution (unsharpened, Å)	2.34	2.66	2.52	2.73	2.64	2.89
FSC 0.143 (unmasked/masked)	2.86 / 2.34	3.16 / 2.66	3.00 / 2.52	3.06 / 2.69	3.11 / 2.59	3.55 / 2.81
Resolution range (local, Å)	2 to 5	2 to 5	2 to 5	2 to 5	2 to 5	2 to 5
Map sharpening *B* factor ($Å^2$)	-35	-30	-35	-35	-5	-20
Map sharpening methods	Relion 3.1	Relion 3.1	Relion 3.1	Relion 3.1	Relion 3.1	Relion 3.1
Non-hydrogen atoms	149,587	146,277	146,882	146,577	154,138	155,151
Protein residues	6,004	5,708	5,740	5,729	6,684	6,878
RNA residues	4,743	4,708,	4,717	4,719	4,720	4,720
Water molecules	50	0	0	0	26	0
Zn^{2+} / Mg^{2+} ions	3/405	1/296	3/368	2/209	3/344	3/197
Ligands	ATP (3), PUT (18), SPD (5)	ATP (2), PUT (11), SPD (4)	ATP (2), PUT (16), SPD (5)	ATP (2), PUT (9), SPD (4)	SCM, GTP, ATP (2), PUT (17), SPD (2)	FUA, GDP, ATP (2), PUT (21), SPD (3)
Refinement package	Phenix	Phenix	Phenix	Phenix	Phenix	Phenix
Resolution cutoff (Å)	2.2	2.6	2.4	2.7	2.5	2.7
**Model-Map scores**						
CC (mask)	0.90	0.78	0.83	0.85	0.88	0.89
CC (volume)	0.89	0.77	0.82	0.84	0.88	0.88
FSC model = 0.143 (unmasked/masked)	2.2/2.1	2.3/2.3	2.2/2.2	2.2/2.3	2.2/2.2	2.4/2.5
**RMSD from ideal values**						
Bond lengths (Å)	0.005	0.007	0.007	0.007	0.010	0.006
Bond angles (°)	0.703	0.957	1.056	1.058	0.845	0.778
**Validation**						
MolProbity score	1.72	1.71	1.70	1.53	2.16	2.31
Clashscore	3.32	2.96	3.20	3.24	4.92	7.08
Cβ deviations	0.14	0.34	0.47	0.57	0.28	0.24
Poor rotamers (%)	3.90	3.19	3.62	2.11	6.47	6.03
CaBLAM outliers (%)	1.90	2.00	1.52	1.28	2.16	2.60
**Ramachandran plot**						
Favored (%)	97.18	96.38	97.05	97.01	95.88	95.32
Allowed (%)	2.82	3.48	2.75	2.95	4.08	4.59
Outliers (%)	0.00	0.14	0.20	0.04	0.05	0.09
**Mean *B* factors** ($Å^2$)						
Protein	91.25	85.72	23.48	15.67	96.41	111.80
RNA	87.85	88.25	33.10	24.32	88.78	102.06
Ligand	62.12	65.09	24.44	21.92	61.16	69.69

Supplementary Tables:

Supplementary Table 2 | Conformational changes within the small subunit (SSU), intramolecular tRNA bend, and mRNA kink during translocation.

Structure	*SSU conformational changes*			*tRNA bending*		*mRNA kink*
	**Body/platform rotation** (°)	**Shoulder rotation** (°)	**Head swivel** (°)	**Deacyl-tRNA^{Phe}** (°)	**Peptidyl-tRNA^{Lys}** (°)	**+3 to +4** (Å)
PRE-C	2.1	4.4	-1.5	20.9	10.6	8.7
PRE-H2*	9.7	4.3	2.7	22.9	22.7	8.9
PRE-H1	11.0	2.0	3.5	17.7	25.5	9.1
INT1	10.8	0.8	5.9	12.9	15.3	8.6
INT2	8.6	2.6	16.1	10.2	8.13	8.2
POST	0	0	0	0	0	7.9

Intramolecular tRNA bend was measured between the anticodon stem loop and the tRNA arms of deacyl-tRNA^{Phe} and peptidyl-tRNA^{Lys}. mRNA kink was measured between the peptidyl- and deacyl-codons during translocation. See Methods for further details. See also Extended Data Fig. 4.

Supplementary Table 3 | Published FRET-pair positions, reported FRET efficiencies, and approximate distances from the points of fluorophore attachment in PRE-C, PRE-H2*, PRE-H1, INT1, INT2, and POST.

Signal	Donor		Acceptor		Publication	FRET Efficiency					
						PRE-C	PRE-H2*	PRE-H1	INT1	INT2	POST
tRNA-tRNA	tRNA^{fMet}	Cy3-s^{4}U8	fMet-Phe-tRNA^{Phe}	Cy5-acp^{3}U47	Munro et al, 2007	~0.55	~0.24	~0.39	-	-	-
	tRNA^{Phe}	Cy3-s^{4}U8	fMet-Phe-Lys-tRNA^{Lys}	Cy5-acp^{3}U47	Wasserman et al, 2016	0.61	0.33		~0.37	~0.6	0.61
					DISTANCE (Å)	~47	~65	~62	~57	~53	~54
tRNA-tRNA	tRNA^{Arg}	Cy3-acp^{3}U47	fMet-Arg-Phe-tRNA^{Phe}	Cy5-acp^{3}U47	Chen et al, 2011	0.69	**0.38**		-	-	0.55
					DISTANCE (Å)	~38	~65	~56	~52	~45	~51
S13-A site	uS13	LD550-ACP-N	fMet-Phe-Lys-tRNA^{Lys}	Cy5-acp^{3}U47	Wasserman et al, 2016		0.13		~0.15		0.34
					DISTANCE (Å)	~89	~97<br>~91*	~87	~89<br>~89.5*	~90	~60
uL11-A site	uL11	Cy3-87	fMet-Arg-Phe-tRNA^{Phe}	Cy5-acp^{3}U47	Chen et al, 2011	0.62	0.34		-	-	0.15
					DISTANCE (Å)	~46	~53	~53	~60	~60	~67
uS13-uL5	uS13	LD550-ACP-N	uL5	LD650-ACP-N	Wasserman et al, 2016	0.76	0.56		~0.5	~0.45	0.76
					DISTANCE (Å)	32	~46	~47	~51	~59	~32
uL9-uS6	bL9	Cy3-(N11C)	bS6	Cy5-(D41C)	Cornish et al, 2008	0.56	-0.4		-	-	-
					DISTANCE (Å)	~55	~67	~67	~66	~64	~56
A site-EF-G	tRNA^{Phe}	Cy3-acp^{3}U47	EF-G	C-SFP-Cy5	Munro et al, 2011	-	-	-	~0.73	~0.55	~0.55
					DISTANCE (Å)	-	-	-	~24	~29	-

Distances are approximate and do not explicitly account for experimental uncertainties in the dye positions and FRET values. *Values are averaged. See also Extended Data Fig. 1h-o. Distance measurements are made between the points of fluorophore attachment.

Supplementary Table 4 | tRNA movement during translocation.

Transition	**Elbow** Position 56 (Å)		**Anticodon** Position 34 (Å)		**Body/anticodon arm** Position 44 (Å)	
	Peptidyl	*Deacyl*	*Peptidyl*	*Deacyl*	*Peptidyl*	*Deacyl*
PRE-C to -H2*	3.1	47.6	5.7	6.3	6.5	17.5
PRE-H2* to -H1	28.2	3.2	3.7	3.0	11.5	2.6
PRE-H1 to INT1	1.2	1.3	3.6	1.0	7.1	2.9
INT1 to INT2	14.8	4.6	5.3	4.5	8.9	7.3
INT2 to POST	0.7	2.7	5.8	3.0	4.1	3.2

Distances between states were measured from the C1’ atom of each designated tRNA nucleotide.

Supplementary Figures

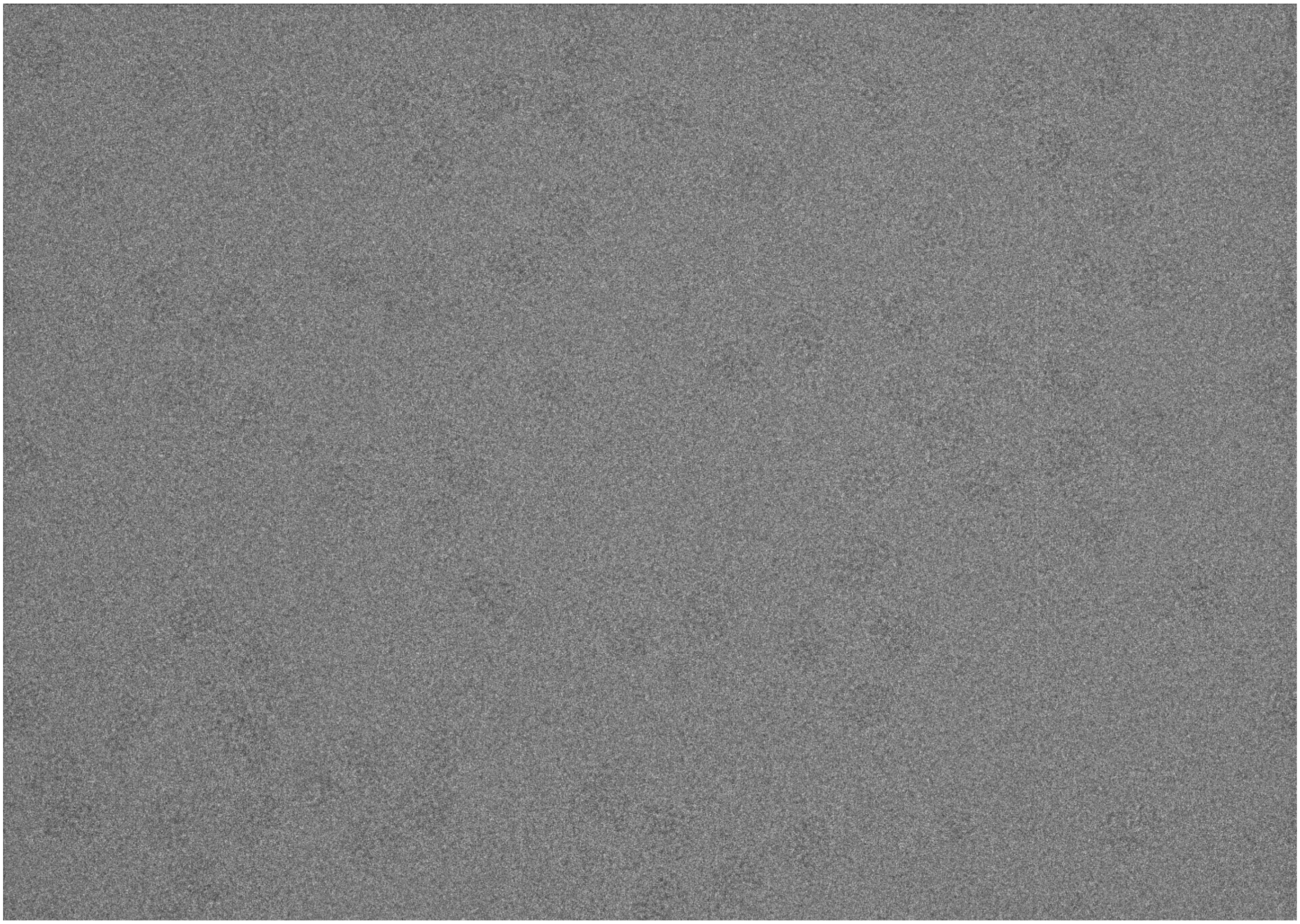

Supplementary Figure 1 | Representative micrograph image from INT1-SPC data collection. Micrograph image showing particle distribution for INT1 data collection. All data collections displayed similar distributions. Image was exported using the Relion 3.1 ‘Display’ function. Pixel size = 0.826 Å/px.
