# Supplementary material for: Structural basis of early translocation events on the ribosome
Source: Nature. 2021 Jul 7;595(7869):741–5. doi: 10.1038/s41586-021-03713-x (PMC8318882; doi:10.1038/s41586-021-03713-x)
Supplement: Supplementary file 2 — Reporting Summary [file 41586_2021_3713_MOESM2_ESM.pdf]

nature research

Corresponding author(s): Scott C. Blanchard

Last updated by author(s): May 30, 2021

Reporting Summary

Nature Research wishes to improve the reproducibility of the work that we publish. This form provides structure for consistency and transparency in reporting. For further information on Nature Research policies, see our Editorial Policies and the Editorial Policy Checklist.

Statistics

For all statistical analyses, confirm that the following items are present in the figure legend, table legend, main text, or Methods section.

n/a	Confirmed	
☐	☒	The exact sample size (*n*) for each experimental group/condition, given as a discrete number and unit of measurement
☐	☒	A statement on whether measurements were taken from distinct samples or whether the same sample was measured repeatedly
☒	☐	The statistical test(s) used AND whether they are one- or two-sided<br>*Only common tests should be described solely by name; describe more complex techniques in the Methods section.*
☒	☐	A description of all covariates tested
☒	☐	A description of any assumptions or corrections, such as tests of normality and adjustment for multiple comparisons
☐	☒	A full description of the statistical parameters including central tendency (e.g. means) or other basic estimates (e.g. regression coefficient) AND variation (e.g. standard deviation) or associated estimates of uncertainty (e.g. confidence intervals)
☒	☐	For null hypothesis testing, the test statistic (e.g. *F*, *t*, *r*) with confidence intervals, effect sizes, degrees of freedom and *P* value noted<br>*Give P values as exact values whenever suitable.*
☒	☐	For Bayesian analysis, information on the choice of priors and Markov chain Monte Carlo settings
☒	☐	For hierarchical and complex designs, identification of the appropriate level for tests and full reporting of outcomes
☒	☐	Estimates of effect sizes (e.g. Cohen's *d*, Pearson's *r*), indicating how they were calculated

Our web collection on statistics for biologists contains articles on many of the points above.

Software and code

Policy information about availability of computer code

Data collection	The time-evolution of FRET signal was then recorded using a home-built total internal reflection based fluorescence microscope at ~0.1 kW/cm2 laser (532 nm) illumination at a time resolution of 40 ms or 400 ms. Donor and acceptor fluorescence intensities were extracted from the recorded movies and FRET efficiency traces were calculated using custom software implemented in MatlabR2015b.<br><br>Cryo-EM data collection was performed using SerialEM software (version 3.7.1) with image shift protocol (9 images were collected with one defocus measurements per 9 holes).
Data analysis	smFRET traces were analyzed using hidden Markov model idealization methods as implemented in the SPARTAN software package (version 3.7.0).<br><br>Motion correction of cryo-EM micrographs was performed on raw super resolution movie stacks and binned 2-fold using MotionCor2 software. CTF parameters were determined using CTFFind4 and refined later in Relion 3.1 and cryoSPARC 3. Prior to particle picking, good micrographs were qualified by power spectrum. Particles were picked within cisTEM 1 and the coordinates were transferred to Relion 3.1.

For manuscripts utilizing custom algorithms or software that are central to the research but not yet described in published literature, software must be made available to editors and reviewers. We strongly encourage code deposition in a community repository (e.g. GitHub). See the Nature Research guidelines for submitting code & software for further information.

Data

Policy information about availability of data

All manuscripts must include a data availability statement. This statement should provide the following information, where applicable:

- Accession codes, unique identifiers, or web links for publicly available datasets
- A list of figures that have associated raw data
- A description of any restrictions on data availability

PDBs and cryo-EM 3D maps for all structures are available through the Protein Data Bank (PDB, www.rcsb.org) and Electron Microscopy Data Bank (EMDB, www.ebi.ac.uk/pdbe/emdb/), respectively (PRE-C, PDB-ID 7N1P, EMD-24120; PRE-H2*, PDB-ID 7N30, EMD-24135; PRE-H1, PDB ID 7N2U, EMD-24133; INT1-SPC, PDB-ID 7N2V, EMD-24134; INT2-FA, PDB-ID 7N2C, EMD-24132; POST, PDB-ID 7N31, EMD-24136). The PDBs that were used as templates for model building (PDB-IDs 4ybb, 5e81, 4wro, 4v9o, 1ctf) are available at www.rcsb.org.

Field-specific reporting

Please select the one below that is the best fit for your research. If you are not sure, read the appropriate sections before making your selection.

☒ Life sciences ☐ Behavioural & social sciences ☐ Ecological, evolutionary & environmental sciences

For a reference copy of the document with all sections, see nature.com/documents/nr-reporting-summary-flat.pdf

Life sciences study design

All studies must disclose on these points even when the disclosure is negative.

Sample size	No statistical methods were used to predetermine sample size. All quantitative experiments were carried out in triplicate and means and standard deviations were calculated. All replicates were included in the analysis. The number of micrographs collected was determined by the number of particles required to achieve a high resolution reconstruction of the target complex.
Data exclusions	No data was excluded from smFRET experiments. Cryo-EM data analysis and classification can be found in Supplementary Information.
Replication	All quantitative measurements were carried out in triplicate and all repeats were included in the analysis, when ap. Cryo-EM data was processed according to standard methods and multiple datasets were collected yielding the same results.
Randomization	The experiments were not randomized as it is not applicable to structural determination and dynamics studies.
Blinding	The investigators were not blinded to allocation during experiments and outcome assessment as none of the experiments involved either human or animal models or group allocation.

Reporting for specific materials, systems and methods

We require information from authors about some types of materials, experimental systems and methods used in many studies. Here, indicate whether each material, system or method listed is relevant to your study. If you are not sure if a list item applies to your research, read the appropriate section before selecting a response.

Materials & experimental systems

n/a	Involved in the study	
☒	☐	Antibodies
☒	☐	Eukaryotic cell lines
☒	☐	Palaeontology and archaeology
☒	☐	Animals and other organisms
☒	☐	Human research participants
☒	☐	Clinical data
☒	☐	Dual use research of concern

Methods

n/a	Involved in the study	
☒	☐	ChIP-seq
☒	☐	Flow cytometry
☒	☐	MRI-based neuroimaging
